# Inhibitory Control of Saccadic Eye Movements and Cognitive Impairment in Mild Cognitive Impairment

**DOI:** 10.3389/fnagi.2022.871432

**Published:** 2022-04-11

**Authors:** Julius Opwonya, Changwon Wang, Kyoung-Mi Jang, Kunho Lee, Joong Il Kim, Jaeuk U. Kim

**Affiliations:** ^1^Digital Health Research Division, Korea Institute of Oriental Medicine, Daejeon, South Korea; ^2^KM Convergence Science, University of Science and Technology, Daejeon, South Korea; ^3^Medical Data Precision Measurement Team, Korea Research Institute of Standards and Science, Daejeon, South Korea; ^4^Gwangju Alzheimer’s Disease and Related Dementias (GARD) Cohort Research Center, Chosun University, Gwangju, South Korea; ^5^Department of Biomedical Science, Chosun University, Gwangju, South Korea; ^6^Dementia Research Group, Korea Brain Research Institute, Daegu, South Korea

**Keywords:** mild cognitive impairment, prosaccade/antisaccade, Go/No-go, frontal/executive function, inhibitory control, self-monitoring

## Abstract

**Background:**

Mild cognitive impairment (MCI) may occur due to several forms of neurodegenerative diseases and non-degenerative conditions and is associated with cognitive impairment that does not affect everyday activities. For a timely diagnosis of MCI to prevent progression to dementia, a screening tool of fast, low-cost and easy access is needed. Recent research on eye movement hints it a potential application for the MCI screening. However, the precise extent of cognitive function decline and eye-movement control alterations in patients with MCI is still unclear.

**Objective:**

This study examined executive control deficits and saccade behavioral changes in patients with MCI using comprehensive neuropsychological assessment and interleaved saccade paradigms.

**Methods:**

Patients with MCI (*n* = 79) and age-matched cognitively healthy controls (HC) (*n* = 170) completed four saccadic eye-movement paradigms: prosaccade (PS)/antisaccade (AS), Go/No-go, and a battery of neuropsychological tests.

**Results:**

The findings revealed significantly longer latency in patients with MCI than in HC during the PS task. Additionally, patients with MCI had a lower proportion of correct responses and a marked increase in inhibition errors for both PS/AS and Go/No-go tasks. Furthermore, when patients with MCI made errors, they failed to self-correct many of these inhibition errors. In addition to the increase in inhibition errors and uncorrected inhibition errors, patients with MCI demonstrated a trend toward increased correction latencies. We also showed a relationship between neuropsychological scores and correct and error saccade responses.

**Conclusion:**

Our results demonstrate that, similar to patients with Alzheimer’s dementia (AD), patients with MCI generate a high proportion of erroneous saccades toward the prepotent target and fail to self-correct many of these errors, which is consistent with an impairment of inhibitory control and error monitoring.

**Significance:**

The interleaved PS/AS and Go/No-go paradigms are sensitive and objective at detecting subtle cognitive deficits and saccade changes in MCI, indicating that these saccadic eye movement paradigms have clinical potential as a screening tool for MCI.

## Introduction

Mild cognitive impairment (MCI) is a syndrome defined as cognitive decline more significant than expected for an individual’s age and education level, but that does not interfere notably with daily life activities (Gauthier et al., 2006). Cognitive or behavioral impairment involves memory, executive function (EF), visuospatial abilities, attention, language, and changes in personality, behavior, or comportment (Petersen, 2004). MCI may be a prodrome to several degenerative, vascular, psychiatric, and medical conditions. It could progress to degenerative diseases, such as Alzheimer’s dementia (AD), frontotemporal dementia (FTD), or dementia with Lewy bodies (DLB); it is also possibly a symptom of non-degenerative conditions, such as vascular cognitive impairment (VCI), major depression, generalized anxiety disorders, uncompensated heart failure, and poorly controlled diabetes mellitus (Petersen, 2016). MCI is characterized as either amnestic MCI (aMCI) or non-amnestic MCI (naMCI), depending on the cognitive domains affected. Both MCI phenotypes could be further divided into single- or multiple-domain MCI depending on the number of cognitive domains affected. The number of domains impacted has crucial implications for determining the degree of the underlying brain pathology, burden of disease, and likelihood of progression to dementia. The annual rate at which MCI progresses to dementia varies between 8 and 15% per year, implying that it is essential to identify and treat patients with MCI (Petersen, 2016). The prevalence of MCI is estimated to be between 15 and 20% in persons 60 years and older, making it a common condition encountered by clinicians. Generally, patients with aMCI eventually develop into AD, and those with naMCI progress into non-AD (Gauthier et al., 2006). Furthermore, since MCI is a clinical diagnosis informed by neuropsychological data, some individuals are never diagnosed; in contrast, other individuals, especially older persons, are often diagnosed late because neuropathologic changes are often present without signs or symptoms (Jack et al., 2018). Sharp distinctions between adults with normal cognition and patients with MCI are complex, and clinical judgment must be used to make these differences (Albert et al., 2011). Therefore, researchers may use biomarker abnormalities that can be detected several years before clinical symptoms appear to aid clinical judgment (Jack et al., 2018). Although these biomarkers refer to those individuals with MCI on the AD spectrum, it is likely that as biomarkers for other disorders evolve, such biomarkers will be matched with a particular phenotype of MCI and increase the ability to predict clinical outcomes.

Several studies suggest that neurodegenerative pathophysiology may be present earlier in vision-related brain structures than in other structures (Albers et al., 2015; Kusne et al., 2017). A previous study found neurofibrillary tangles and neuritic plaques in the occipital cortex of healthy adult subjects and patients with MCI (McKee et al., 2006). These findings provide histopathological evidence that MCI caused by degenerative disease processes disrupts the visual system much earlier than previously thought and thus substantiates the use of visual biomarkers in early disease detection. Many studies have also reported significant differences in structural and functional visual measures between patients with MCI and those with normal cognition (Javaid et al., 2016; Chan et al., 2019), with some tests being capable of detecting MCI (Galetta et al., 2017). Eye tracking, especially saccadic eye movement (SEM) evaluation, is beneficial in determining the disease stage in patients with minor motor dysfunction and cognitive deficits (Anderson and MacAskill, 2013). There are two main types of SEMs: visually guided saccades and voluntary (or volitional) saccades. A visually guided saccade is an involuntary positioning reaction to a new event in the field of vision. In contrast, voluntary saccades result from purposeful activity in various paradigms, such as antisaccade (AS), memory-guided saccade, and predictive saccade paradigms. More extensive reliance on higher-level executive control during volitional saccades results in increasingly complex brain stimulation patterns. The network involved in saccade generation includes subcortical and cortical regions (Leigh and Kennard, 2004; Pierrot-Deseilligny et al., 2004). Central to these executive control functions is the ability to appropriately select behaviorally advantageous actions and withhold or suppress inappropriate actions in a given behavioral context (response inhibition) because they interfere with completing motor and cognitive goals. Response inhibition is an essential EF implemented by the prefrontal cortex (PFC). Various cortical subregions, such as the ventrolateral prefrontal cortex (VLPFC), presupplementary motor area (pre-SMA), dorsolateral prefrontal cortex (DLPFC), lateral orbitofrontal and anterior cingulate (ACC) areas, play vital roles in inhibitory behavior control, and both brain hemispheres are involved in inhibitory cognitive function (Bokura et al., 2001; Simmonds et al., 2008; Chikazoe, 2010). Pertinently, inhibitory control is a heterogeneous construct (Barkley, 1997; Nigg, 2000; Aron, 2011), and therefore, researchers use a range of paradigms, such as AS and Go/No-go, to assess specific aspects of this function.

In AS trials, at least two steps are necessary: inhibition of the reflexive response for a visually guided saccade to the target and the reversal of the stimulus location into a voluntary motor command to look in the direction opposite from the stimulus. AS trials activate the oculomotor network more than visually guided trials (e.g., PS and Go trials) and may also recruit additional brain areas, such as the DLPFC and ACC, which are unnecessary in visually guided trials. The AS task encompasses various cognitive processes, such as decision making, working memory, goal-oriented behavior, learning, and attention (Jamadar et al., 2013). The traditional Go/No-go task design involves only two stimuli: a “Go” stimulus and a “No-go” stimulus. In Go/No-go tasks, a “Go” stimulus (or stimuli) requires executing a response, and a “No-go” stimulus (or stimuli) requires withholding a response (response inhibition). Typically, the task is weighted toward Go stimuli to build up a prepotent tendency to respond, thereby increasing the inhibitory effort necessary to withhold responding to No-go stimuli successfully (Simmonds et al., 2008). Performance of a Go/No-go task requires the recruitment of various cognitive components, including working memory, goal-oriented behavior, and attention (Chikazoe, 2010). Studies employing Go/No-go and AS tasks have shown very similar activation patterns, suggesting that these tasks inherently test the same cognitive processes (i.e., response inhibition) (Chikazoe, 2010). Additionally, response inhibition is vital to isolate and identify as it relates to self-regulatory processes in behavior and behavior changes (Simmonds et al., 2008). Crawford et al. (2005) showed that patients with AD have impaired inhibitory control and error correction that exceed the effects of normal aging; these impairments are related to the severity of dementia. To the best of our knowledge, no study has shown the extent of inhibitory control using both PS/AS and Go/No-go paradigms in patients with MCI. To examine the specific self-monitoring process, we distinguished between the inhibition errors that were corrected and uncorrected. We defined inhibition errors as the combined sum of corrected and uncorrected errors. Uncorrected-inhibition errors consisted of the trials in which the eye was captured inappropriately by the target, but participants did not make an error-corrected saccade.

Previous work has shown that PS/AS paradigms could distinguish controls from patients with MCI using latency and error saccade parameters (Opwonya et al., 2021). However, most studies have had few participants and used a blocked administration approach while conducting eye-tracking tasks. It is not well established whether the outcomes between these two methods are similar since the administration approach (block vs. interleaved) affects saccade latencies and errors; the interleaved approach produces larger PS/AS differences in error rates, while block administration produces larger latency differences (Zeligman and Zivotofsky, 2017). To determine the saccade parameter differences between subjects with MCI and controls, we tested patients in a series of interleaved saccadic paradigms: PS/AS and Go/No-go. To date, most eye-tracking studies have examined Western and educated populations. A drawback of this constraint on the studied population is that it limits the understanding; hence, they cannot be generalized to other populations since eye movements vary across different cultural and ethnic groups (Rayner et al., 2007; Knox et al., 2012). Here, we studied saccadic behavior patterns in East Asia to bridge this gap.

In summary, this study aimed to broaden the current knowledge of saccade behavior and executive control deficits related to eye-movement control in patients with MCI using interleaved saccade paradigms and neuropsychological assessment. Additionally, this paper discusses specific features of eye-movement control–saccadic latency and inhibitory control in patients with MCI and how these features relate to the typical effects of healthy aging in native Koreans. Considerable knowledge of the neural basis of eye movements and the overlap of structures implicated in inhibition with those suggested to be dysfunctional in patients with MCI make inhibitory oculomotor outcomes potential biomarkers for screening and identifying MCI. The analysis of eye-tracking measures provides information on the cognitive and neural mechanisms involved in the volitional control of behavior. We speculate that brain changes occurring in MCI patients may influence their saccade behavior, providing the potential for cost-effective and straightforward objective measures to assess executive control deficits in patients with MCI.

## Materials and Methods

### Participants

In this study, 274 subjects participated, and we excluded nine subjects because they were diagnosed with AD. In addition, we excluded 16 more subjects from the analysis who were difficult to measure due to color blindness, poor vision, or sagging eyelids, or because they did not pass the preliminary exercise and calibration test before this trial. We divided the participants into cognitively healthy age-matched control (HC) and MCI groups. All the participants were examined by a clinical interview, which included the assessment of the clinical dementia rating (CDR). HC participants had a CDR score of 0. They had a normal range of cognitive function and good general health with no evidence of brain atrophy, white matter changes, multiple lacunae, infarction, or other focal brain lesions on magnetic resonance imaging (MRI) scans. MCI participants met the Petersen criteria (Petersen, 2004) and had a CDR score of 0.5. Their neuropsychological test z scores were below -1.5 on at least one of five domain tests according to age-, education-, and sex-specific norms. The HC group had 170 subjects (72 males and 98 females), and their mean age was 71.5 ± 6.2 years; the MCI group had 79 subjects (38 males and 41 females), and their mean age was 73.3 ± 7.7 years (see Table 1). All participants provided written informed consent, and the Institutional Review Board of Chonnam National University Hospital approved the study (IRB No. CNUH-2019-279).

**TABLE 1:** Participant’s demographic information and neuropsychological score.

	HC	MCI			
		
	(*n* = 170)	(*n* = 79)	*T* statistic	*P* value	Effect size (95% CI)
**Demographic**					
Sex, M/F	72/98	38/41		0.475^a^	
Age, yr	71.5 (6.2)	73.3 (7.7)	−1.831	0.069^b^	−0.259 (−0.527, 0.010)
Education, yr	11.0 (4.5)	10.5 (4.7)	0.761	0.447^c^	0.104 (−0.163, 0.371)
**Neuropsychological**					
K-MMSE	27.5 (1.8)	25.8 (3.3)	4.709	**<0.001^b^**	0.702 (0.416, 0.984)
Attention	9.8 (2.0)	8.3 (1.9)	5.363	**<0.001^c^**	0.733 (0.457, 1.008)
Language	0.2 (0.3)	−0.1 (0.5)	4.79	**<0.001^b^**	0.706 (0.420, 0.988)
Visuospatial	0.6 (0.4)	0.2 (0.9)	4.001	**<0.001^b^**	0.616 (0.332, 0.898)
Memory	0.3 (0.6)	−0.5 (0.7)	9.038	**<0.001^b^**	1.283 (0.970, 1.592)
Frontal	0.2 (0.6)	−0.4 (0.7)	6.922	**<0.001^b^**	0.970 (0.677, 1.260)

*CI, confidence interval; HC, healthy control; MCI, mild cognitive impairment.*

*Values are shown as the mean (standard deviation). Statistically significant test results are highlighted in boldface.*

*^a^χ^2^-test.*

*^b^Welch t-test.*

*^c^Student’s t-test.*

### Eye-Tracking Recordings

Saccadic eye movements were recorded using a Tobii Pro spectrum system (Tobii Technology AB, Stockholm, Sweden) with a 300 Hz sampling rate and processed with Tobii Pro Lab (ver. 1.118). The distance between the participant and the monitor was approximately 65 cm. Furthermore, we used a desk with an adjustable chin and forehead rest to maintain an appropriate angle between the eye-tracking monitor and each subject’s gaze.

**FIGURE 1:**
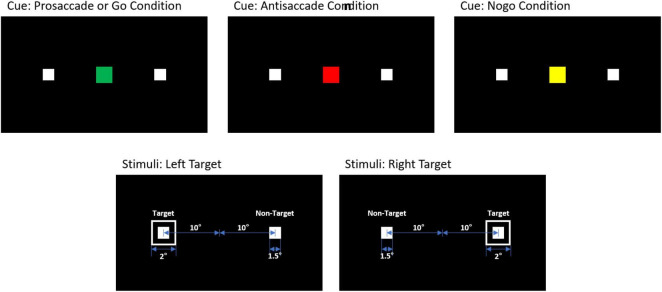
Cue and target stimuli for PS/Go, AS, and No-go trials.

We presented visual stimuli on the Tobii Pro Spectrum screen (EIZO FlexScan EV2451) with 1,920 × 1,080 pixel (52.8 × 29.7 cm) resolution. Participants were asked to perform PS or AS and Go or No-go tasks in response to cues presented on the screen (see Figure 1). Each trial began with the appearance of a fixation point (1.5° diameter) for 500 ms on a black background in the center of the screen. The trial condition was revealed via the cue color (2° diameter): green for the PS and Go condition, red for the AS condition, and yellow for the No-go condition. After a cue period of 800 ms, the stimuli disappeared for 200 ms before the target appeared (1° diameter), 10° from the center. The target remained on the screen for 1,500 ms. We inserted a gap period between the cue and the peripheral stimulus to examine preparatory processes and induce more directional errors in the AS condition.

**FIGURE 2:**
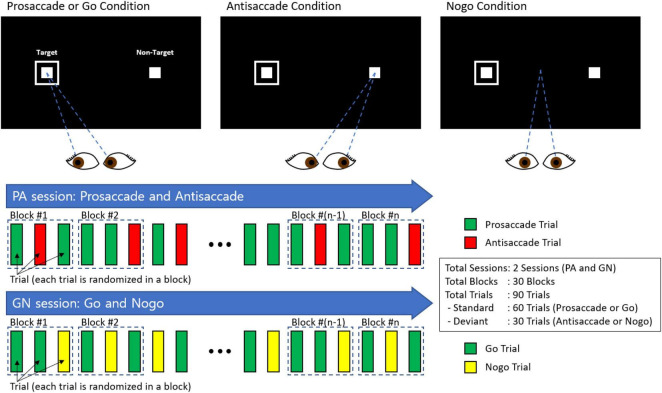
PS/AS and Go/No-go paradigms.

The design of our experiment included a combination of the two sessions: one session comprising interleaved PS and AS trials and the other session comprising Go and No-go trials (see Figure 2). Each session had 30 blocks with randomized and counterbalanced trials, with each block containing 2 trials of PS/Go and 1 trial of AS/No-go.

Saccadic eye movement data preprocessing was performed using MATLAB (R2019b; MathWorks Inc., Natick, MA, United States). We designed the AOI (area of interest) algorithm to classify the correct/incorrect responses and set the gaze variation threshold value at 1°. We defined correct responses when the gaze variation was 1°, and the gaze was fixed in the set AOI. According to our area of interest (AOI) decision algorithm, we classified a total of six responses: invalid responses, correct responses, anticipatory errors, omissions, self-corrected-inhibition errors, and uncorrected-inhibition errors; these responses were identified using the definitions put forward by Crawford et al. (2005). We defined all errors as the sum of anticipatory errors, omissions, self-corrected errors (PS/Go trials), corrected-inhibition errors (AS/No-go trials), and uncorrected-inhibition errors (AS/No-go). The specific method of classifying the six responses was as follows. First, the fixation duration was calculated by checking whether or not the saccade movement was within the AOI and whether the gaze variation was within the threshold. The invalid responses were classified as responses with less than 80% validity. The correct responses were defined when onset time was between 80 and 500 ms, minimum fixation duration (minFD) was greater than 100 ms, and maximum gaze variation (maxGV) of fixation for the target less than 1°. The response was classified as an anticipatory error when the fixation duration was less than 80 ms. The omission errors were defined when no saccade movement occurred within 500 ms. The self-corrected-inhibition errors were classified when the gaze shifted within 400 ms from the wrong gaze direction to the correct gaze direction, and the gaze variation was within 1° at the same time. The uncorrected-inhibition error was defined when the condition of minimum fixation duration less than 100 ms and maximum gaze variation within 1° was not satisfied.

### Neuropsychological Battery

In this study, we used the Korean version of the Mini-Mental State Examination (K-MMSE) and the current version of the Seoul Neuropsychological Screening Battery (SNSB II) to evaluate cognitive function (Park, 1989; Kang et al., 1997, 2003). The SNSB II is one of South Korea’s most commonly used neuropsychological screening batteries for assessing cognitive functioning in patients with MCI and dementia. The SNSB II consists of five cognitive domain scores: attention, language, memory, visuospatial, and frontal/EFs. The estimated completion time of the whole battery is 1.5–2 h.

### Statistical Analyses

All statistical analyses were performed in RStudio version 4.1.2 for Windows. We carried out *t* tests, and Pearson’s *r* correlations analyses. We examined the relationships of neuropsychological domains and saccade responses using partial correlation adjusted for age, sex, and years of education. Partial correlation analyses were performed separately between five neuropsychological domains and saccade parameters for participants in the MCI and control groups. For all between-group comparisons, *p* value < 0.05 was considered significant and we used Cohen’s *d* to compute effect size estimates (Cohen, 2013). For inclusion in statistical analyses, the subjects needed at least 50% valid trials for each condition (e.g., for PS: at least 30 valid trials out of 60). Thus, the final sample size varied between different eye-tracking outcome measures according to specific inclusion criteria for the eye-tracking tasks.

## Results

### Saccade Responses

#### Prosaccade

**FIGURE 3:**
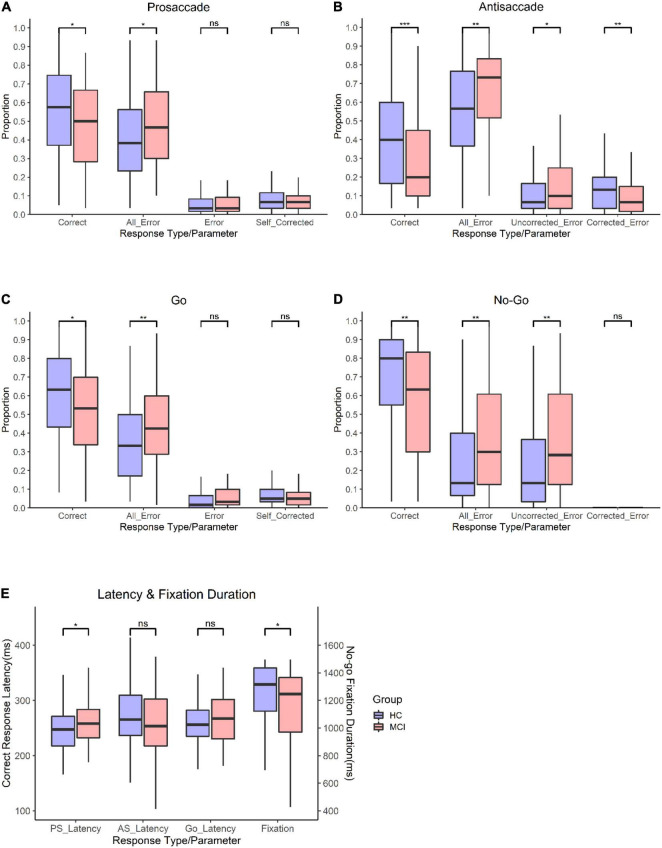
Oculomotor responses in controls and patients with MCI. **(A)** Proportion for prosaccade trials. **(B)** Proportion for antisaccade trials. **(C)** proportion for go trials. **(D)** Proportion for no-go trials. **(E)** Latency for PS, AS, Go trials, and proportion and fixation duration for no-go trials. **Significance level *p* < 0.001; *significance level *p* < 0.05; n.s., not significant.

Figure 3 shows the results of the PS/AS and Go/No-go saccade responses. In the PS task, patients with MCI were slower to initiate eye movements than HC [*t*(247) = −2.28, *p* = 0.024]. In addition, subjects with MCI had a smaller proportion of correct saccades [*t*(247) = 2.27, *p* = 0.024] and generated more errors [*t*(247) = −2.27, *p* = 0.024] than HC. There was a similar distribution of self-corrected errors [*t*(247) = 0.911, *p* = 0.363] among participants in both groups (see Supplementary Table 1).

#### Antisaccade

In the AS paradigm, patients with MCI had a smaller proportion of correct responses (t (226) = 3.33, *p* = 0.01) than HC. Moreover, subjects with MCI had a higher frequency of errors [*t*(226) = −3.15, *p* = 0.002], corrected a smaller proportion of errors [*t*(226) = 2.62, *p* = 0.009], and had a higher proportion of uncorrected errors [*t*(226) = −2.36, *p* = 0.019] than controls. There was no significant difference in the latency of correct AS responses [*t*(226) = 1.12, *p* = 0.265].

#### Go

In the Go task, patients with MCI had a lower proportion of correct responses than HC [*t*(246) = 2.68, *p* = 0.008] and had a higher frequency of all errors [*t*(246) = −2.81, *p* = 0.005]. The latency of correct responses [*t*(246) = −1.65, *p* = 0.099] and self-corrected errors [*t*(246) = 1.14, *p* = 0.254] did not differ significantly between the groups.

#### No-Go

In the No-go task, patients with MCI had a smaller proportion of correct responses [*t*(233) = 3.06, *p* = 0.002] than HC. Additionally, subjects with MCI had shorter fixation durations [*t*(233) = 2.36, *p* = 0.019], generated a higher proportion of errors [*t*(233) = −3.07, *p* = 0.002], and had a higher proportion of uncorrected errors [*t*(233) = −3.12, *p* = 0.002] than controls. However, the corrected-inhibition errors [*t*(233) = 0.453, *p* = 0.964] did not differ between subjects with MCI and HC.

### Correlation

#### Prosaccade/Antisaccade and Neuropsychological Tests

**FIGURE 4:**
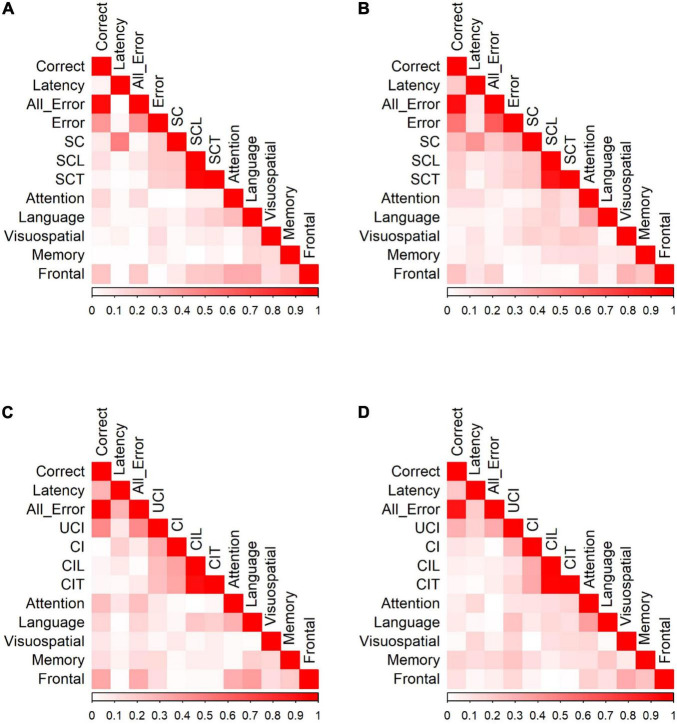
Correlation of PS/AS responses and neuropsychological tests for **(A)** PS responses by HC. **(B)** PS responses by MCI patients. **(C)** AS responses by HC. **(D)** AS responses by MCI patients. Data were presented with the absolute values of Pearson correlation coefficients; exact values and *p-*values are presented in Supplementary Table 2 (HC) and Supplementary Table 3 (MCI). SC, self-corrected errors; SCL, self-corrected latency; SCT, self-corrected time; UCI, uncorrected-inhibition errors; CI, corrected inhibition errors; CIL, corrected inhibition latency; CIT, corrected inhibition time. Weak correlations, near 0, are in white while those nearing 1 are in red, portraying strong correlations. Pearson’s correlations are in absolute values.

We calculated Pearson correlation coefficients to identify significant relationships between saccadic eye movements and the raw scores of five cognitive domains for participants in each group. Figure 4 shows the correlations between PS/AS responses and SNSB II domain scores for normal controls and patients with MCI (detailed statistical values are presented in Supplementary Table 2 for HC and Supplementary Table 3 for MCI). In control participants, partial correlation analysis demonstrated the presence of statistically significant positive correlations between EF and the number of correct responses (*r* = 0.234, *p* = 0.002) and revealed negative correlations between EF and all errors (*r* = −0.218, *p* = 0.005), between EF and self-corrected latency (*r* = −0.211, *p* = 0.006), and between EF and time-to-correct errors (*r* = −0.225, *p* = 0.003). Negative correlations were also found between language and time-to-correct errors (*r* = −0.184, *p* = 0.017). No significant correlations were found in the attention, visuospatial domains, or memory domains. In participants in the MCI group, partial correlation highlighted the presence of a significant positive correlation between EF and correct latency (*r* = 0.238, *p* = 0.039). We did not find significant correlations in the attention, language, visuospatial, and memory domains.

In the AS paradigm, oculomotor responses correlated with a number of neuropsychological measures. In HC, partial correlation analysis demonstrated the presence of statistically significant positive correlations between EF and the number of correct AS responses (*r* = 0.353, *p* < 0.001) and significant negative correlations between EF and all errors (*r* = −0.332, *p* < 0.001). There were also significant correlations between attention and the number of correct AS responses (*r* = 0.255, *p* = 0.001), attention and all errors (*r* = −0.252, *p* = 0.002), language and correct responses (*r* = 0.169, *p* = 0.036), language and all errors (*r* = −0.166, *p* = 0.040), language and corrected-inhibition latency (*r* = −0.224, *p* = 0.005), and language and time taken to correct inhibition (*r* = −0.188, *p* = 0.019). In participants in the MCI group, adjusting for covariates diminished all significant correlations.

#### Go/No-Go Tasks and Neuropsychological Tests

**FIGURE 5:**
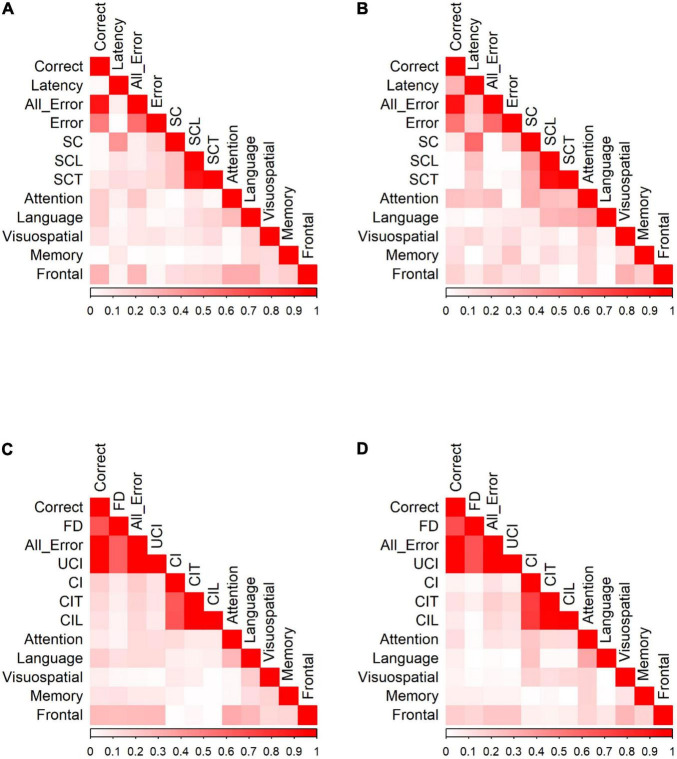
Correlation of Go/No-Go responses and neuropsychological tests for **(A)** Go responses by HC. **(B)** Go responses by MCI patients. **(C)** No-Go responses by HC. **(D)** No-Go responses by MCI patients. Data were presented with the absolute values of Pearson correlation coefficients; exact values and *p* values are presented in Supplementary Table 2 (HC) and Supplementary Table 3 (MCI). FD, fixation duration, SC, self-corrected errors, SCL, self-corrected latency, SCT, self-corrected time, UCI, uncorrected-inhibition errors, CI, corrected inhibition errors, CIL, corrected inhibition latency, and CIT, corrected inhibition time. Weak correlations, near 0, are in white while those nearing 1 are in red, portraying strong correlations. Pearson’s correlations are in absolute values.

We calculated Pearson correlation coefficients to identify significant relationships between saccadic eye movements and the raw scores of five cognitive domains for participants in each group. Figure 5 shows the correlation of Go/No-go saccadic parameters and SNSB II domain scores for HC and patients with MCI (detailed statistical values are presented in Supplementary Table 2 for HC and Supplementary Table 3 for MCI). In control participants, partial correlation analysis demonstrated statistically significant positive correlations between attention and the number of correct responses (*r* = 0.191, *p* = 0.013) and negative correlations between attention and all errors (*r* = −0.214, *p* = 0.005). Similarly, there were positive correlations between language and the number of correct responses (*r* = 0.192, *p* = 0.013) and negative correlations between language and time-to-correct (*r* = −0.167, *p* = 0.031). In addition, EF demonstrated statistically significant positive correlations with the number of correct responses (*r* = 0.295, *p* < 0.001) and negative correlations with all errors (*r* = −0.285, *p* < 0.001), self-corrected latency (*r* = −0.154, *p* = 0.047), and time-to-correct (*r* = −0.161, *p* = 0.038). In the MCI group, partial correlation highlighted significant relationships between attention and latency of correct responses (*r* = 0.245, *p* = 0.035), between attention and all errors (*r* = −0.256, *p* = 0.028), and between attention and the number of self-corrected errors (*r* = −0.329, *p* = 0.004). There was also a significant negative relationship between language and self-corrected latency (*r* = −0.280, *p* = 0.016) and between language and time-to-correct errors (*r* = −0.306, *p* = 0.008). No significant correlations were found in the visuospatial, memory, and EF domains.

HC showed significant correlations between SNSB II domains and the number of correct responses, fixation duration, all errors, uncorrected-inhibition errors, and corrected-inhibition errors in the no-go task. After adjusting for covariates, most significant correlations diminished, with the strongest correlations observed between EF and correct responses (*r* = 0.272, *p* < 0.001), between EF and fixation duration (*r* = 0.260, *p* < 0.001), between EF and all errors (*r* = −0.270, *p* < 0.001), and between EF and uncorrected-inhibition errors (*r* = −0.275, *p* < 0.001). We also observed significant correlations between language and the correct number of responses (*r* = 0.195, *p* = 0.014). In MCI group, the number of correct responses, fixation duration, all error, uncorrected-inhibition errors, and corrected-inhibition errors all had significant correlations with several SNSB II domain scores. Performing a partial correlation to account for the possible influence of demographic factors, such as age, sex, and years of education on these relationships eliminated all the correlations.

## Discussion

In this study, our main objectives were to increase the current knowledge of saccade behavior and executive control deficits related to eye-movement control and to examine how the saccade parameters in patients with MCI relate to the typical features of cognitively healthy aging. We examined visually guided saccades and voluntary/volitional saccadic eye responses between patients with MCI and HC using interleaved PS/AS and Go/No-go paradigms. We also investigated the link between saccadic eye movement parameters and several neuropsychological tests frequently used during MCI screening.

Previous eye-tracking studies have shown a close relationship between cognitive processes and fixation duration, with longer fixations associated with greater attentional focus (Rayner, 2009; Henderson et al., 2015). Fixation duration represents the relative engagement with the object, i.e., the greater the average fixation duration, the greater the level of engagement. Our study found that the MCI group had a shorter fixation duration than controls, which may indicate poorer attentional focus in patients with MCI than healthy controls.

In our study’s visually guided tasks (PS and Go), the MCI group had a lower proportion of correct saccades than healthy controls had in all valid trials. There are several possible explanations for this result. First, this outcome could indicate that subjects with MCI have deficits in the involuntary control of saccades resulting from dysfunction of the neural circuitry. The visually guided saccade generation network includes subcortical and cortical regions affected by MCI due to degeneration (Braak and Braak, 1995). Another possible explanation for the poorer performance is that patients have cognitive control deficits, and some cognitive processes such as attention and learning influence performance on visually guided saccades (Hutton, 2008). The AS paradigm requires a volitional saccade away from the target plus inhibition of a visually guided saccade. In contrast, the No-go task requires only the inhibition of a visually guided saccade. The proportion of correct responses in AS and No-go tasks revealed a significant difference between the MCI group and controls. This significantly lower number of correct responses in the MCI group may suggest a deficit of voluntary saccadic control in patients with MCI as volitional control tasks have increasingly complex brain stimulation patterns that recruit additional neural regions such as the prefrontal cortex not activated in visually guided tasks (McDowell et al., 2008). Coupled with the executive control deficits seen in patients with MCI, they may be unable to cope with these additional demands and hence the poorer performance when compared to healthy controls.

In the current study, when we compared the latency of correct saccades in the MCI group to that of HC in the visually guided paradigms, we found that patients had longer latencies when initiating saccades in the PS task. In contrast, the MCI group had similar latencies with controls in the Go task. These differences can partly be explained by the weaker attention-demanding nature of the Go/No-go paradigm, enabling MCI participants to automatically orient their gaze to the target with similar reaction times as controls. It is also fundamental to note that during the interleaved PS/AS trials, the subjects do not know the trial type and, aware of the possibility of an AS, engage proactive inhibitory control to tamper with the involuntary PS. The longer latencies in patients with MCI during PS suggest that during the gap period, attentional disengagement which is regulated by the superior colliculus (SC), occurs at a slower rate in patients. In other words, the inhibition of the fixation neurons is more prolonged, and the activation of the saccade neurons to aid the beginning of a successive saccade is slower in patients with MCI than in HC. The results point to the likelihood that the subcortical oculomotor network is affected in the early stages of the disease (Braak and Braak, 1995). Notably, when we compared the AS latency of correct saccades in patients with MCI to HC, there was no significant difference between the groups. We previously showed that in PS/AS block administration, PS latency did not reliably distinguish between patients with MCI and age-matched controls, but AS latency was significantly different between the groups (Opwonya et al., 2021). The differences in saccade latency suggest that latency differences between patients with MCI and HC are more pronounced in the PS interleaved and AS block modes, indicating a possible effect of the mode of administration (blocks vs. randomly interleaved).

In this study, in both visually guided paradigms, the MCI group committed a higher number of errors than HC. When we assessed all errors by subtype, the proportion of self-corrected errors, self-corrected latency, and time-to-correct erroneous saccade showed little effect of disease, as there was no significant difference between the groups. The proportion of inhibition errors in AS trials is a robust measure of inhibitory control, with fewer errors signifying better response inhibition (Munoz and Everling, 2004). The primary dependent measure in Go/No-go tasks is the error commission rate, i.e., making a “Go” response on “No-go” trials. This study indicates that patients with MCI had higher inhibition errors in both No-go and AS trials. In the Go/No-go paradigm, patients with MCI produced approximately 10% more inhibition errors than controls. In the PS/AS paradigm, patients with MCI produced approximately 5% more inhibition errors than controls. Pertinently, Go/No-go task performance is also subject to a high cognitive load. It depends on the ability to switch between inhibitory and movement modes on a trial-by-trial basis, contributing to the high error rates in this task (Crawford et al., 2005). Overall, there was a higher proportion of inhibition errors in the MCI group, probably resulting from alterations in the PFC circuitry leading to changes in the input to other oculomotor regions involved in the initiation and execution of voluntary saccades (Leigh and Kennard, 2004). The findings observed in this study mirror those of previous studies that have examined the effect of inhibitory control in patients with MCI and early AD (Crawford et al., 2005; Alichniewicz et al., 2013; Heuer et al., 2013; Holden et al., 2018; Wilcockson et al., 2019).

When people commit errors, the deviation from intended behavior may require a minimal behavioral adjustment, or it may entail reassessing an entire behavioral plan. An efficient error-monitoring system should be able to evaluate the significance of an error, in addition to detecting that an error has occurred. In both the AS and No-go tasks, the MCI group had more uncorrected-inhibition errors than HC. Additionally, in the AS task, the MCI group corrected significantly fewer errors than HC. The weaker attention-demanding nature of the No-go paradigm revealed some preservation of inhibitory capacity in both groups of participants as there was no significant difference in corrected errors. A possible explanation for these findings might be that some errors, such as rapidly corrected saccades, may not even reach the level of consciousness (Nieuwenhuis et al., 2001). We believe that patients with MCI are more susceptible to not correcting errors committed due to alterations in the self-monitoring and error correction network, which recruits the PFC and ACC regions. This finding supports existing research highlighting the relationship between uncorrected-inhibition errors and dysregulation of the self-monitoring and error correction network (Crawford et al., 2013; Peltsch et al., 2014; Wilcockson et al., 2019). Overall, these findings are consistent with the dysfunction of the error-monitoring processes in patients with MCI.

The present study results determined the correlation between specific saccade parameters and neuropsychological measures. We found weak to moderate correlations across various measures in line with previous studies (Heuer et al., 2013; Yang et al., 2013; Peltsch et al., 2014; Chehrehnegar et al., 2019; Wilcockson et al., 2019; Polden et al., 2020). Some of the correlations were likely related to demographic factors (e.g., age, sex, and years of education) because controlling for them diminished the effects, especially for the MCI subjects. In general, saccade parameters correlated most strongly with executive function, language, and attention test scores, which index the functioning of these cognitive domains. The correlation between executive function, language, and attention scores and visually guided saccades is noteworthy because these saccades might be considered a reflexive movement toward a visual target onset that would not place demands on higher-order cognitive processes. However, the findings suggest that in interleaved trials, aware of the likelihood of an AS or No-go, the participants engage proactive inhibitory control processes that permit saccade planning to be adjusted based on the cue’s cognitive processing, indicating the appropriate response for each trial. Consequently, the visually guided saccades are not purely involuntary, and cognitive control processes are involved in executing these saccades.

Furthermore, our study found a significant positive correlation between neuropsychological scores and correct responses and a negative correlation with error responses. Specific brain regions are implicated in the control of these eye responses. The parietal eye field (PEF), which projects to the SC, plays a vital role in triggering reflexive saccades and is essential in preparing for correct saccades (Leigh and Kennard, 2004). Other brain areas vital for correct and error responses include the supplementary eye field (SEF) involved in sequencing and planning saccades and the DLPFC crucial for inhibiting unwanted saccades (Leigh and Kennard, 2004). Negative correlations between cognitive measures and error responses in the MCI group may suggest that neurodegenerative changes in areas responsible for high-level cognitive functions and saccade execution are progressing in parallel. Also, the negative correlations may indicate an association between early cognitive decline and inhibitory impairment. These results build on existing evidence of a relationship between executive functions and error responses (Heuer et al., 2013; Peltsch et al., 2014; Koçoğlu et al., 2021). Overall, our data offers evidence for a direct relationship between cognitive measures of executive function, language, attention, and correct and error responses modulated by higher cortical regions.

Limitations of our study are as following. Eye-movement control may be influenced by culture and ethnicity factors (Knox et al., 2012; Polden et al., 2020), and we examined saccades in only native Korean participants, which may limit the generalizability of our findings. Secondly, our results were derived from a single eye-tracking recording, and MCI patients may revert to normal or progress to other conditions. Therefore, studying how the saccade parameters change over time is worth investigating. Future longitudinal studies will help to determine the clinical implications of these findings.

## Conclusion

Our data provided a detailed description of saccade performance changes in MCI subjects who underwent rigorous neuropsychological assessment. This study provided further evidence that in the interleaved design, visually guided saccades can be modified based on cognitive processing of the cue indicating the appropriate response for each trial and shows a relationship between EF and saccade error responses. Additionally, we highlighted the importance of the mode of administration (blocks vs. randomly interleaved) on saccade latency between patients with MCI and HC, substantially adding to the understanding of the effects of administration mode on saccade behavior. Furthermore, the results of this study indicate that PS/AS and Go/No-go paradigm data may reveal distinctive deficits in latency, inhibitory control, and error monitoring in MCI patients compared to these aspects in cognitively healthy age-matched controls.

Taken together, the evidence from this study suggested that performance in the PS/AS and Go/No-go paradigms is sensitive and objective for detecting subtle cognitive deficits. Combining these results with future longitudinal studies that track which MCI patients progress to other conditions has strong potential for clinical application.

## Data Availability Statement

The original contributions presented in the study are included in the article/Supplementary Material, further inquiries can be directed to the corresponding author/s.

## Ethics Statement

The studies involving human participants were reviewed and approved by the Institutional Review Board of Chonnam National University Hospital (IRB No. CNUH-2019-279). The patients/participants provided their written informed consent to participate in this study.

## Author Contributions

JUK, JIK, CW, K-MJ, KL, and JO made conceptualization, methodology, software, validation, investigation, writing—review and editing, and project administration contributions. JO, CW, and JIK made formal analysis, data curation, and writing—original draft preparation contributions. JUK conducted supervision and funding acquisition. All authors read and agreed to the published version of the manuscript.

## Conflict of Interest

The authors declare that the research was conducted in the absence of any commercial or financial relationships that could be construed as a potential conflict of interest.

## Publisher’s Note

All claims expressed in this article are solely those of the authors and do not necessarily represent those of their affiliated organizations, or those of the publisher, the editors and the reviewers. Any product that may be evaluated in this article, or claim that may be made by its manufacturer, is not guaranteed or endorsed by the publisher.
