# Supplementary material for: Inhibitory Control of Saccadic Eye Movements and Cognitive Impairment in Mild Cognitive Impairment
Source: Front Aging Neurosci. 2022 Apr 11;14:871432. doi: 10.3389/fnagi.2022.871432 (PMC9038187; doi:10.3389/fnagi.2022.871432)
Supplement: Supplementary file 1 [file Table_1.docx]

**Appendix**

**Table. 1** Comparison of eye movement data between HC and MCI

|  |  | HC | MCI |  |  |  |
| --- | --- | --- | --- | --- | --- | --- |
| Task | Parameters | Mean (SD) | Mean (SD) | T statistic | P value | Effect size (95% CI) |
| Prosaccade  (HC = 170, MCI = 79) | Correct | 32.7 (15) | 28.2 (13.7) | 2.272 | **0.024**^a^ | 0.309 (0.041, 0.577) |
|  | Latency | 247.9 (40.9) | 260.5 (39.9) | -2.277 | **0.024**^a^ | -0.31 (-0.578, -0.041) |
|  | All Errors | 24.7 (14) | 28.9 (13.1) | -2.268 | **0.024**^a^ | -0.309 (-0.577, -0.04) |
|  | SC | 4.5 (3.6) | 4.1 (3) | 0.911 | 0.363^a^ | 0.124 (-0.143, 0.391) |
|  | SCL | 363.6 (153.5) | 386.1 (151.1) | -1.085 | 0.279^a^ | -0.148 (-0.415, 0.12) |
|  | SCT | 180.4 (82.1) | 193 (87.6) | -1.101 | 0.272^a^ | -0.15 (-0.417, 0.117) |
| Antisaccade  (HC = 157, MCI = 71) | Correct | 12.3 (8) | 8.6 (7) | 3.326 | **0.001**^a^ | 0.476 (0.191, 0.759) |
|  | Latency | 270.3 (60.1) | 260.5 (63.7) | 1.117 | 0.265^a^ | 0.16 (-0.121, 0.44) |
|  | All Errors | 16.9 (7.5) | 20.1 (6.6) | -3.151 | **0.002**^a^ | -0.451(-0.734, -0.167) |
|  | UI | 3.4 (4.4) | 5 (5.1) | -2.233 | **0.027**^b^ | -0.328 (-0.611, -0.044) |
|  | CI | 4.1 (3.4) | 2.9 (2.9) | 2.623 | **0.009**^a^ | 0.375 (0.092, 0.657) |
|  | CIL | 371.5 (165.6) | 335.6 (215.2) | 1.25 | 0.214^b^ | 0.187 (-0.095, 0.468) |
|  | CIT | 180.4 (86) | 166.8 (107) | 0.938 | 0.350^b^ | 0.139 (-0.142, 0.42) |
| Go  (HC = 170, MCI = 78) | Correct | 36.3 (14.1) | 31 (15.2) | 2.678 | **0.008**^a^ | 0.366 (0.096, 0.636) |
|  | Latency | 259.4 (38) | 268.5 (45.1) | -1.553 | 0.123^b^ | -0.219 (-0.488, 0.051) |
|  | All Errors | 20.9 (12.8) | 25.9 (13.3) | -2.81 | **0.005**^a^ | -0.384 (-0.654, -0.114) |
|  | SC | 4.3 (3.5) | 3.7 (3.3) | 1.143 | 0.254^a^ | 0.156 (-0.112, 0.425) |
|  | SCL | 362.6 (143.2) | 340.5 (173.6) | 0.98 | 0.329^b^ | 0.139 (-0.13, 0.407) |
|  | SCT | 180.9 (78.6) | 173.1 (96.1) | 0.629 | 0.530^b^ | 0.089 (-0.179, 0.357) |
| No-Go  (HC = 163, MCI = 72) | Correct | 20.9 (8.5) | 17.1 (9.3) | 3.059 | **0.002**^a^ | 0.433 (0.152, 0.712) |
|  | FD | 1246.9 (233.8) | 1163.9 (278.5) | 2.363 | **0.019**^a^ | 0.334 (0.055, 0.613) |
|  | All Errors | 7.6 (7.9) | 11.1 (8.9) | -3.067 | **0.002**^a^ | -0.434 (-0.714, -0.153) |
|  | UI | 7.3 (7.8) | 10.9 (8.8) | -2.979 | **0.003**^b^ | -0.431 (-0.713, -0.148) |
|  | CI | 0.3 (0.7) | 0.3 (0.6) | 0.045 | 0.964^a^ | 0.006 (-0.271, 0.284) |
|  | CIL | 30.9 (82) | 43.2 (93) | -1.017 | 0.310^a^ | -0.144 (-0.421, 0.134) |
|  | CIT | 28.3(71.6) | 39.8(87) | -1.055 | 0.293^a^ | -0.149(-0.427,0.129) |
| HC: Healthy control, MCI: mild cognitive impairment, SD: Standard deviation, FD: Fixation duration, SC: Self-corrected errors, SCL: Self-corrected latency, SCT: Self-corrected time, UCI: Uncorrected inhibition errors, CI: Corrected inhibition errors, CIL: Corrected inhibition latency, and CIT: Corrected inhibition time. ^a^ Student's t-test, ^b^ Welch t-test. | | | | | | |

**Table. 2** Pearson partial correlations of eye movement data and SNSB domains for HC

| HC | | | | | | |
| --- | --- | --- | --- | --- | --- | --- |
|  |  | SNSB Domains | | | | |
| Task | ET variables | Attention | Language | Visuospatial | Memory | Frontal |
| Prosaccade | Correct | 0.149 (0.054) | 0.081 (0.299) | 0.027 (0.732) | 0.011 (0.888) | 0.234 (0.002) |
|  | Latency | 0.024 (0.755) | -.024 (0.754) | -.052 (0.504) | -.011 (0.887) | 0.01 (0.899) |
|  | All_Error | -0.136 (0.08) | -.025 (0.745) | -0.006 (0.94) | -.008 (0.918) | -0.218 (0.005) |
|  | Error | 0.01 (0.902) | 0.076 (0.327) | 0.131 (0.092) | -.081 (0.296) | 0.013 (0.865) |
|  | SC | -.001 (0.993) | 0.045 (0.563) | -.023 (0.769) | 0.025 (0.748) | -0.078 (0.314) |
|  | SCL | -0.057 (0.462) | -.127 (0.101) | -.067 (0.388) | 0.004 (0.961) | -0.211 (0.006) |
|  | SCT | -0.055 (0.482) | -.184 (0.017) | -.076 (0.326) | 0.045 (0.56) | -0.225 (0.003) |
| Antisaccade | Correct | 0.255 (0.001) | 0.169 (0.036) | 0.093 (0.253) | 0.128 (0.113) | 0.353 (< .001) |
|  | Latency | 0.101 (0.212) | 0.009 (0.909) | 0.031 (0.707) | 0.051 (0.532) | 0.012 (0.878) |
|  | All_Error | -0.252 (0.002) | -0.166 (0.04) | -.096 (0.235) | -0.137 (0.09) | -.332 (< .001) |
|  | UCI | -0.09 (0.265) | -.086 (0.289) | -.031 (0.699) | -.143 (0.078) | -0.126 (0.119) |
|  | CI | -0.021 (0.801) | -.039 (0.629) | 0.083 (0.308) | 0.026 (0.75) | 0.019 (0.816) |
|  | CIL | 0.000982 (0.99) | -.224 (0.005) | -.032 (0.692) | 0.07 (0.386) | 0.024 (0.77) |
|  | CIT | 0.036 (0.66) | -.188 (0.019) | -.065 (0.426) | 0.068 (0.402) | 0.02 (0.808) |
| Go | Correct | 0.191 (0.013) | 0.192 (0.013) | 0.117 (0.134) | 0.012 (0.88) | 0.295 (< .001) |
|  | Latency | 0.065 (0.403) | 0.034 (0.661) | 0.049 (0.527) | 0.086 (0.268) | 0.045 (0.559) |
|  | All_Error | -0.214 (0.005) | -.097 (0.211) | -0.09 (0.245) | -.008 (0.916) | -.285 (< .001) |
|  | Error | 0.053 (0.494) | 0.029 (0.71) | 0.102 (0.19) | 0.022 (0.773) | -0.039 (0.615) |
|  | SC | -0.01 (0.895) | 0.035 (0.658) | 0.073 (0.345) | -.004 (0.964) | -0.133 (0.086) |
|  | SCL | -0.091 (0.244) | -.123 (0.112) | -.099 (0.203) | -.031 (0.695) | -0.154 (0.047) |
|  | SCT | -0.032 (0.685) | -.167 (0.031) | -.139 (0.072) | -0.042 (0.59) | -0.161 (0.038) |
| No-go | Correct | 0.087 (0.274) | 0.195 (0.014) | 0.067 (0.397) | 0.107 (0.176) | 0.272 (< .001) |
|  | FD | 0.04 (0.616) | 0.124 (0.119) | 0.032 (0.689) | 0.118 (0.137) | 0.26 (< .001) |
|  | All_Error | -0.143 (0.072) | -.145 (0.068) | -.025 (0.754) | -.085 (0.285) | -0.27 (< .001) |
|  | UCI | -0.131 (0.098) | -.141 (0.076) | -.017 (0.828) | -.082 (0.305) | -.275 (< .001) |
|  | CI | -0.14 (0.076) | -.065 (0.417) | -.082 (0.305) | -.049 (0.542) | 0.006 (0.944) |
|  | CIL | -0.076 (0.341) | -0.05 (0.533) | -.057 (0.471) | -.006 (0.945) | -0.036 (0.655) |
|  | CIT | -0.078 (0.326) | -.065 (0.412) | -.013 (0.873) | -.008 (0.918) | -0.005 (0.951) |
| Values presented are Pearson correlation coefficients and their corresponding p-value. | | | | | | |

**Table. 3** Pearson partial correlations of eye movement data and SNSB domains for MCI group

| MCI | | | | | | |
| --- | --- | --- | --- | --- | --- | --- |
|  | SNSB Domains | | | | | |
| Task | ET variables | Attention | Language | Visuospatial | Memory | Frontal |
| Prosaccade | Correct | 0.129 (0.269) | 0.051 (0.663) | 0.036 (0.761) | 0.049 (0.675) | 0.238 (0.039) |
|  | Latency | 0.134 (0.252) | -.051 (0.663) | -0.106 (0.366) | -0.09 (0.437) | -0.102 (0.38) |
|  | All_Error | -.064 (0.587) | 0.033 (0.781) | 0.037 (0.755) | -0.041 (0.727) | -0.184 (0.112) |
|  | Error | -.036 (0.762) | -.084 (0.475) | 0.107 (0.362) | 0.029 (0.802) | 0.005 (0.966) |
|  | SC | 0.055 (0.639) | 0.141 (0.228) | 0.176 (0.131) | -0.043 (0.71) | -0.035 (0.764) |
|  | SCL | 0.189 (0.105) | 0.177 (0.129) | -0.154 (0.188) | -0.12 (0.3) | -0.026 (0.827) |
|  | SCT | 0.124 (0.287) | 0.1 (0.394) | -0.209 (0.072) | -0.134 (0.25) | -0.018 (0.878) |
| Antisaccade | Correct | 0.073 (0.558) | 0.075 (0.546) | 0.021 (0.864) | 0.172 (0.16) | 0.116 (0.346) |
|  | Latency | 0.152 (0.219) | 0.032 (0.798) | -0.159 (0.198) | 0.132 (0.282) | 0.036 (0.771) |
|  | All_Error | -.001 (0.993) | 0.006 (0.962) | 0.054 (0.667) | -0.157 (0.201) | -0.065 (0.596) |
|  | UCI | 0.109 (0.38) | -.237 (0.053) | 0.167 (0.176) | -0.23 (0.059) | -0.152 (0.216) |
|  | CI | -.113 (0.361) | 0.089 (0.474) | 0.003 (0.978) | 0.125 (0.308) | 0.043 (0.725) |
|  | CIL | 0.133 (0.284) | 0.151 (0.224) | 0.122 (0.326) | 0.06 (0.626) | -0.003 (0.98) |
|  | CIT | 0.16 (0.196) | 0.12 (0.335) | 0.142 (0.252) | 0.113 (0.358) | 0.009 (0.945) |
| Go | Correct | 0.245 (0.035) | -.031 (0.794) | 0.112 (0.343) | -0.131 (0.264) | 0.179 (0.123) |
|  | Latency | 0.218 (0.063) | 0.007 (0.953) | -0.147 (0.211) | -.000 (0.999) | -0.08 (0.493) |
|  | All_Error | -.256 (0.028) | 0.062 (0.6) | -0.077 (0.513) | 0.096 (0.415) | -0.181 (0.119) |
|  | Error | -.017 (0.883) | -.079 (0.504) | 0.135 (0.252) | 0.22 (0.058) | 0.117 (0.318) |
|  | SC | -.329 (0.004) | 0.094 (0.426) | 0.059 (0.618) | -0.046 (0.696) | -0.136 (0.244) |
|  | SCL | -0.25 (0.032) | -0.28 (0.016) | 0.086 (0.467) | -0.138 (0.238) | 0.045 (0.704) |
|  | SCT | -.232 (0.046) | -.306 (0.008) | 0.025 (0.836) | -0.071 (0.544) | 0.011 (0.927) |
| No-go | Correct | 0.127 (0.298) | 0.061 (0.622) | 0.045 (0.713) | -0.058 (0.633) | 0.198 (0.102) |
|  | FD | -.015 (0.901) | 0.003 (0.981) | 0.015 (0.901) | -0.056 (0.647) | 0.169 (0.166) |
|  | All_Error | -.111 (0.362) | -.019 (0.875) | -0.032 (0.795) | 0.048 (0.696) | -0.229 (0.059) |
|  | UCI | -.097 (0.426) | -.004 (0.971) | -0.021 (0.863) | 0.048 (0.697) | -0.226 (0.061) |
|  | CI | -.228 (0.059) | -.238 (0.051) | -0.172 (0.161) | 0.008 (0.946) | -0.057 (0.641) |
|  | CIL | -.141 (0.247) | -.021 (0.866) | -0.122 (0.323) | -0.031 (0.798) | -0.052 (0.669) |
|  | CIT | -.132 (0.281) | -.024 (0.846) | -0.147 (0.233) | 0.004 (0.971) | -0.074 (0.544) |
| Values presented are Pearson correlation coefficients and their corresponding p-value. | | | | | | |
